# Successful Treatment of Glans Tumescence Dysfunction Caused by Abnormal Venous Communication Between the Great Saphenous Vein and the Deep Dorsal Vein: A Case Report

**DOI:** 10.1155/criu/6933520

**Published:** 2026-07-17

**Authors:** Alessandro Zucchi, Fabio Pezzoni, Filippo Dazzi, Matteo Pacini, Antonio Luigi Pastore, Manfredi Sequi, Fabio Maria Valenzi, Fabrizio Ildefonso Scroppo

**Affiliations:** ^1^ Department of Translational Research and New Technologies in Medicine and Surgery, University of Pisa, Pisa, Italy, unipi.it; ^2^ University of Milan, Milan, Italy, unimi.it; ^3^ Urology Unit, Department of Medico-Surgical Sciences and Biotechnologies, Faculty of Pharmacy and Medicine, Sapienza University of Rome, Latina, Italy, uniroma1.it; ^4^ Urology Department, Andrology Unit, Villa Aprica Clinical Institute, Como, Italy

**Keywords:** case report, cavernosography, deep dorsal vein, erectile dysfunction, glans tumescence, great saphenous vein, venous leak

## Abstract

**Background:**

Penile venous abnormalities are an uncommon cause of erectile dysfunction and are rarely implicated in isolated glans tumescence impairment. We report what may be the first successful surgical treatment of glandular tumescence dysfunction caused by an anomalous venous communication between the deep dorsal vein of the penis and the left great saphenous vein.

**Case Presentation:**

A 42‐year‐old man presented with lifelong inadequate glans tumescence and mild erectile maintenance difficulty (International Index of Erectile Function‐5 score, 18). Physical examination and hormonal evaluation were unremarkable. Dynamic penile color Doppler ultrasonography after intracavernosal prostaglandin E1 demonstrated normal cavernosal arterial inflow and preserved cavernous veno‐occlusive function but showed markedly increased flow in the deep dorsal vein despite rigid shaft erection and persistent poor glans engorgement. Dynamic cavernosography identified an isolated anomalous collateral vessel connecting the deep dorsal vein to the left great saphenous vein, with no additional venous leakage sites. Surgical correction consisted of ligation and excision of the anomalous collateral vein, excision of the deep dorsal vein through an anterograde penile skin degloving approach, and selective ligation of cavernous veins.

**Conclusions:**

The patient reported complete restoration of glans tumescence at 1 month, sustained at 1 year, with improvement of the International Index of Erectile Function‐5 score from 18 to 23. This case highlights the diagnostic value of cavernosography in selected patients with discordant findings between shaft rigidity and glans engorgement and suggests that targeted surgical correction may be effective when a highly localized venous shunt is identified.

## 1. Background

Penile erection depends on a coordinated interaction between arterial inflow and restriction of venous outflow. When the veno‐occlusive mechanism fails, the penis cannot maintain the pressures required for full rigidity, resulting in venous leak–related erectile dysfunction [1, 2]. Although venous leak is a recognized cause of erectile impairment, the exact anatomy of the drainage pathways involved, especially when uncommon collateral routes are present, remains incompletely characterized [2, 3].

Earlier cavernosographic studies showed that venous outflow may occur not only through the deep dorsal and cavernous veins, but also through less common aberrant pathways toward extrapenile venous systems [3, 4]. Among these, drainage toward the great saphenous vein (GSV) appears to be unusual but physiologically relevant because it may provide a low‐resistance channel that interferes with intracavernosal pressure build‐up [3, 5].

We describe a patient with lifelong inadequate glans tumescence and mild erectile maintenance difficulty in whom dynamic cavernosography revealed an isolated abnormal communication between the deep dorsal vein (DDV) and the left GSV. This appears to be a rare and previously unreported anatomical cause of glans tumescence dysfunction. The specific incidence of venogenic erectile dysfunction (venous leak) caused by abnormal communications with the GSV is considered very low and estimated to be around 4%–9% [6]. Moreover, the case is noteworthy because the venous anomaly offered a clear anatomical explanation for the clinical presentation and because surgical correction was followed by durable symptomatic improvement.

## 2. Case Presentation

A 42‐year‐old man presented with lifelong inadequate glans tumescence and a slight erectile dysfunction mainly represented by an occasionally difficulty in erection maintenance. His International Index of Erectile Function‐5 (IIEF‐5) score was 18. He reported preserved libido, ejaculation, and orgasm, and no major couple distress. He denied diabetes, hypertension, dyslipidemia, pelvic surgery, penile trauma, Peyronie′s disease, lower urinary tract symptoms, or known endocrine disorders. Morning erections were present but consistently characterized by suboptimal glans engorgement.

Previous treatment with on‐demand phosphodiesterase type‐5 inhibitors (tadalafil 10–20 mg) achieved satisfactory shaft rigidity but did not improve glans tumescence. He had not previously used intracavernosal agents.

Physical examination of the external genitalia was normal, with no palpable plaques, penile curvature, meatal abnormalities, or corpus spongiosum abnormalities. Serum testosterone, luteinizing hormone, follicle‐stimulating hormone, estradiol, and prolactin concentrations were within normal limits.

**Figure 1:**
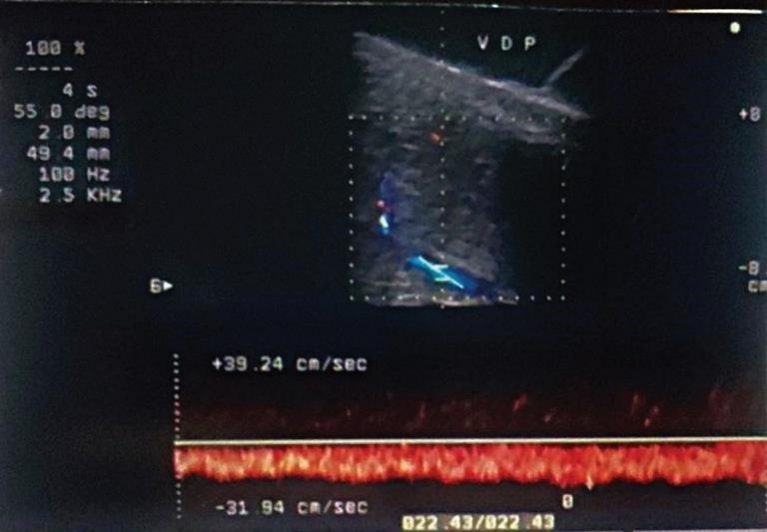
Normal cavernosal artery inflow on dynamic penile color Doppler ultrasonography.

**Figure 2:**
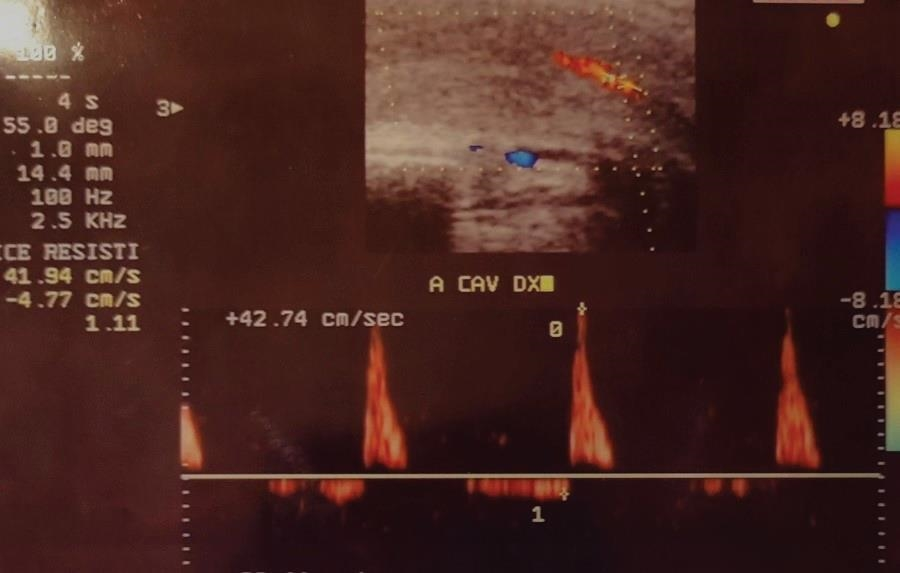
Increased flow velocity in the deep dorsal vein on Doppler evaluation.

Dynamic penile color Doppler ultrasonography was performed after intracavernosal injection of prostaglandin E1 (20 ug). Cavernosal arterial inflow was normal, with peak systolic velocity greater than 35 cm/s and end‐diastolic velocity less than 5 cm/s, consistent with preserved cavernous veno‐occlusive function. A rigid shaft erection persisted for approximately 45 min. However, glans tumescence remained incomplete. In contrast with the otherwise normal hemodynamic profile, Doppler interrogation of the DDV demonstrated markedly increased venous flow velocity (> 30 cm/s) (Figures 1 and 2).

Arterial inflow to the glans was additionally assessed using continuous‐wave Doppler with an 8‐MHz pencil probe directed at distal dorsal and bulbourethral arterial flow; findings were normal.

**Figure 3:**
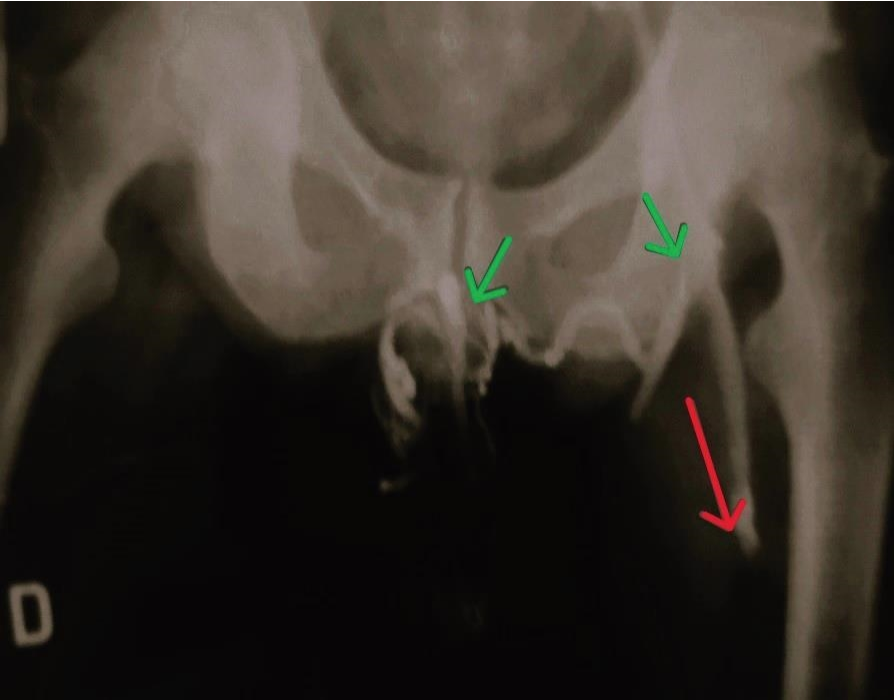
Dynamic cavernosography showing the anomalous collateral vessel connecting the deep dorsal vein and the left great saphenous vein. Red arrows indicate the anomalous vessel at its origin and confluence. Green arrow indicates the first competent saphenous valve.

Because shaft rigidity was preserved despite poor glans tumescence, dynamic cavernosography (spongiosography) was performed. Under local anesthesia, an endo‐glans approach was used with a 21‐gauge butterfly needle. After intracavernosal papaverine (40 mg), 50 mL of warmed iodinated contrast medium was infused at a controlled rate under fluoroscopic guidance. Early opacification of the DDV occurred within seconds, followed by visualization of a single anomalous collateral vessel of slightly increased caliber coursing dorsolaterally and connecting the DDV to the left GSV. Contrast progression stopped at the first competent saphenous valve in the proximal thigh, producing a distinct filling stop (Figure 3). No additional leakage was observed through the cavernous veins, corpus spongiosum, or glans venous plexuses.

These findings supported the diagnosis of a highly localized veno‐occlusive abnormality involving an aberrant DDV‐GSV communication. The venographic pattern suggested a suction effect toward the femoro‐iliac venous system at the expense of deep dorsal and cavernous venous outflow, thereby providing an anatomical correlate for the patient′s lifelong glans tumescence deficit.

Surgical correction was undertaken with ligation and excision of the anomalous collateral vein, isolation and complete excision of the DDV through an anterograde penile skin degloving approach, and selective ligation of the circumflex veins. The operative goal was to eliminate the pathological high‐flow venous shunt toward the low‐pressure extracavernous venous system and reinforce the penile veno‐occlusive mechanism. The specific anomalous venous communication was precisely localized during surgery on the basis of dynamic cavernosography.

In this specific case, the choice to use open surgery rather than endovascular procedures—such as vessel sclerotherapy—could have led to potential complications, such as the migration of the sclerosant agent into the deep venous system. On the other hand, the incision for the isolation of the DDV and a small counterincision at the crural level presented no difficulties in isolating, ligating, and removing the entire anomalous vessel with the aid of a standard vascular tunneler.

There were no postoperative complications, and the procedure was performed by a vascular surgeon/andrologist. In this specific case, the choice to use open surgery rather than endovascular procedures—such as vessel sclerotherapy—could have led to potential complications, such as the migration of the sclerosant agent into the deep venous system, especially given the normal competence of the saphenofemoral junction itself. On the other hand, the incision for the isolation of the DDV and a small counterincision at the crural level presented no difficulties in isolating, ligating, and removing the entire anomalous vessel with the aid of a standard vascular tunneler.

At 1 month after surgery, the patient reported complete restoration of glans tumescence. This result remained stable at 1 year of follow‐up. The IIEF‐5 score improved from 18 to 23, corresponding to near‐normal erectile function. It remains obvious that the IIEF score depends on the overall quality of the erection and that the ligation of the DDV and the anomalous collateral vessel can contribute to improving the IIEF‐5 score by reducing the pathological venous outflow. However, specifically, the improvement in glandular tumescence after surgical treatment—the primary objective of the case presented—must be considered.

## 3. Discussion and Conclusions

This case illustrates an unusual venous mechanism underlying erectile dysfunction with predominant impairment of glans tumescence. In the present patient, the discrepancy between normal cavernosal arterial inflow, preserved shaft rigidity, and persistently poor glans engorgement prompted further investigation beyond routine duplex assessment. Cavernosography proved decisive by identifying a single abnormal communication between the DDV and the GSV.

Classic work by Reiss [4] described the role of spongiosography in delineating penile venous drainage, whereas Shabsigh et al. [3] documented both common and aberrant venous leakage routes in patients with normal arterial inflow. Their observations included uncommon collateral drainage toward the saphenous and femoral systems, supporting the pathophysiological plausibility of the present finding [3]. Schramek and Waldhauser [7] also reported isolated glans insufficiency related to venous leakage, reinforcing the concept that impaired glans tumescence may have a specific venous substrate rather than simply reflecting generalized erectile dysfunction.

From a clinical perspective, this case suggests two practical messages. First, persistent poor glans tumescence in the presence of adequate shaft rigidity should raise suspicion for a selective venous abnormality [7]. Second, dynamic cavernosography may still have an important role in highly selected patients when noninvasive hemodynamic findings are discordant with the clinical picture [3, 4].

The favorable postoperative course observed here suggests that targeted surgical correction may be effective when a discrete anatomical shunt can be demonstrated. Nevertheless, conclusions must remain cautious because the evidence derives from a single case, and broader conclusions regarding efficacy or indications cannot be drawn without additional reports.

**Nomenclature**
DDV: deep dorsal vein
GSV: great saphenous vein
IIEF‐5: International Index of Erectile Function‐5
PGE1: prostaglandin E1

## Author Contributions

Conceptualization: Matteo Pacini, Alessandro Zucchi, Filippo Dazzi, and Fabrizio Ildefonso Scroppo. Manuscript drafting: Filippo Dazzi and Alessandro Zucchi. Manuscript revision: Alessandro Zucchi, Matteo Pacini, Filippo Dazzi, Fabrizio Ildefonso Scroppo, Fabio Pezzoni, Manfredi Sequi, Antonio Luigi Pastore, and Fabio Maria Valenzi. Data collection: Fabrizio Ildefonso Scroppo and Fabio Pezzoni. Data analysis: Fabrizio Ildefonso Scroppo and Fabio Pezzoni. Supervision: Fabrizio Ildefonso Scroppo, Fabio Pezzoni, and Alessandro Zucchi. Alessandro Zucchi and Fabio Pezzoni have contributed equally to the manuscript.

## Funding

Open access publishing was facilitated by the Universita degli Studi di Pisa, as part of the Wiley‐CRUI‐CARE agreement.

## Disclosure

All authors read and approved the final manuscript.

## Ethics Statement

Written informed consent for publication of this case report and the accompanying images was obtained from the patient.

## Consent

Written informed consent for publication of this case report and the accompanying images was obtained from the patient.

## Conflicts of Interest

The authors declare no conflicts of interest.

## Author Biographies

**Alessandro Zucchi** and **Filippo Dazzi** are affiliated with the Department of Translational Research and New Technologies in Medicine and Surgery, University of Pisa, Pisa, Italy.

**Fabio Pezzoni** is an andrologist and vascular surgeon affiliated with the University of Milan, Milan, Italy.

**Manfredi Sequi**, **Antonio Luigi Pastore**, and **Fabio Maria Valenzi** are affiliated with the Urology Unit, Department of Medico‐Surgical Sciences and Biotechnologies, Faculty of Pharmacy and Medicine, Sapienza University of Rome, Latina, Italy.

**Fabrizio Ildefonso Scroppo** is affiliated with the Urology Department, Andrology Unit, Villa Aprica Clinical Institute, Como, Italy.

**Matteo Pacini** is affiliated with the Urology Unit, Department of Translational Research and New Technologies in Medicine and Surgery, University of Pisa, Pisa, Italy.

## Data Availability

All data generated or analyzed during this study are included in this published article. Further information may be requested to the corresponding author.
